# AhR Antagonist Promotes Differentiation of Papillary Thyroid Cancer *via* Regulating circSH2B3/miR-4640-5P/IGF2BP2 Axis

**DOI:** 10.3389/fphar.2021.795386

**Published:** 2021-12-23

**Authors:** Ri Sa, Meiliang Guo, Danyan Liu, Feng Guan

**Affiliations:** ^1^ Department of Nuclear Medicine, The First Hospital of Jilin University, Changchun, China; ^2^ Department of Dermatology, Shanghai Jiao Tong University Affiliated Sixth People’s Hospital, Shanghai, China; ^3^ Department of Radiology, The First Hospital of Jilin University, Changchun, China

**Keywords:** AhR antagonist, papillary thyroid cancer, differentiation, circSH2B3, IGF2BP2

## Abstract

Abnormally high expression of aryl hydrocarbon receptor (AhR) has been implicated in dedifferentiation of radioiodine-refractory papillary thyroid cancer (RR-PTC). This study aimed to evaluate the differentiation effect of AhR antagonist in PTC, and to explore the potential mechanism of it. Results showed that AhR antagonists promoted differentiation of PTC, as shown as increase in ^125^I uptake and Na/I symporter (NIS) expression level. CircRNA microarray in K1 cells treated with StemRegenin 1(SR1) revealed that hsa_circ_0006741 (circSH2B3) was down-regulated in SR1 treated K1 cells. Downregulation of circSH2B3 increased ^125^I uptake and NIS expression levels. CircSH2B3 acted as an endogenous sponge of hsa-miR-4640-5p and modulated IGF2BP2 expression. IGF2BP2 overexpression induced dedifferentiation of PTC, while silencing IGF2BP2 accelerated differentiation of PTC cells. Rescue studies showed that the dedifferentiation activity of AhR was modulated by the circSH2B3/miR-4640-5p/IGF2BP2 axis. Our findings confirmed for the first time that AhR antagonists promote differentiation of PTC via inhibiting the circSH2B3/miR-4640-5p/IGF2BP2 axis, offering a novel therapeutic approach and a potential marker for differentiation of PTC.

## Introduction

According to the 2020 global cancer statistics, thyroid cancer is one of the most common endocrine malignancies in the worldwide, with responsible for approximately 58,600 cases, ranking in ninth place for incidence in 2020 (Sung et al., 2021). Papillary thyroid cancer (PTC) is the most prevalent subtype of thyroid cancer, accounting for 80% of all thyroid cancers. Radioiodine (^131^I) therapy has been the standard of care for unresectable ^131^I -avid metastatic PTC, and ^131^I uptake is a good prognostic marker (Ahn, 2016). Although most patients do well, the biology of PTC is extremely diverse, ranging from well ^131^I-responsive indolent lesions to ^131^I-refractory locally advanced or metastatic disease. The mean life span of ^131^I refractory disease is less than 5 years and the 10-years-survival rate is often less than 10% (Durante et al., 2006).

Differentiation therapy followed by ^131^I is a promising alternative therapy for radioiodine refractory thyroid cancer (RR-PTC). To date, several differentiation therapies including retinoic acid (Schmutzler and Köhrle, 2000), tyrosine-kinase inhibitors (Oh et al., 2020), histone deacetylase inhibitors (Jang et al., 2015), peroxisome proliferator-activated receptor (PPAR)-*γ* agonists etc. (Park et al., 2005), have been adopted to enhance Na/I symporter (NIS) function to reverse the dedifferentiation of RR-PTC and support ^131^I therapy. It is feasible, theses differentiation strategies have already brought significant clinical benefits (Buffet et al., 2020). However, only a limited number of patients got benefit from these therapeutic strategies, necessitating the development of other new agents to aid the differentiation of PTC, as well as further research to look into the underlying potential mechanism of dedifferentiation of PTC.

More recent work suggests that the aryl hydrocarbon receptor (AhR), a cytosolic ligand-activated transcription factor, is expressed at aberrantly low or high levels in several malignancies, integrating with promotion or inhibition of cell differentiation (Zhao et al., 2019). Thus, AhR agonists and antagonists have been used to mediate cell differentiation. In melanoma, AhR agonists, for example, can activate the AhR-dependent resistant program to induce dedifferentiation, whereas AhR antagonists can abrogate deleterious AhR sustained-activation to overcome dedifferentiation and drug resistance (Corre et al., 2018). In leukemic stem cells, the AhR pathway is suppressed (Ly et al., 2019). By down-regulating the stem cell transcription factor Oct4, AhR enhanced retinoic acid induced differentiation of myeloblastic leukemia cells (Bunaciu and Yen, 2011). Given that AhR contributes to cell differentiation in several malignancies, there is a strong likelihood that AhR might be involved in differentiation of PTC.

Herein, we confirmed the differentiation effect of AhR antagonists in PTC. *Via* circRNA microarray, we discovered a previously undescribed circRNA hsa_circ_0006741 (termed as circSH2B3) contributing to AhR antagonist induced differentiation of PTC. Therefore, this study was designed to explore the role of circSH2B3 in AhR antagonist induced differentiation of PTC and elucidate the underlying mechanism.

## Material and Methods

### Cell Culture and Agents

A normal thyroid follicular epithelial cell line (Nthy-ori 3-1) and PTC cell lines (K1 and BCPAP) were purchased from the Chinese Academy of Sciences. All cell lines had been tested and authenticated by DNA analysis. The cells were cultured in RPMI 1640 supplemented with 20% fetal bovine serum (FBS), 100 units/ml of penicillin, and 100 µg/ml streptomycin at 37°C and 5% CO2. AhR antagonists, StemRegenin 1 and CH-223191, were purchased from Selleck.

### Plasmid Construction and Transfection

Human short hairpin RNA (shRNA) and overexpressed circSH2B3 were designed and synthesized by Genomeditech (Shanghai, China). Hsa-miR-4640-5p mimics or inhibitors, as well as their controls were synthesized by Genomeditech (Shanghai, China). Cell transfection was performed using Lipofectamine 2000 (Invitrogen, NY, United States) according to the manufacturer’s instruction. shRNAs and overexpressed of IGF2BP2 lentivirus plasmid (HanBio, Shanghai, China) were transfected into the polybrene treated PTC cells. In the presence of 5 µg/ml puromycin, the transfection lentivirus content was according to the individual multiplicity of infection. Empty vector was used as a negative control. qRT-PCR was used to assess transfection efficiency.

### Cell Viability Assay

Cells were grown in the presence of different doses of StemRegenin 1 (0, 62.5, 125, 250, 500, 1,000, 2000, 4,000 nM) or CH-223191 (0, 7.8, 15.6, 31.3, 62.5, 125, 250, 500 nM) for 48 h. 10 μL of CCK8 reagent (Yeasen, Shanghai, China) was added, incubate for 2 h, and the absorbance measured at 450 nm. Using GraphPad Prism v. 8.0 (GraphPad Software, CA, United States), the half-maximal inhibitory concentration (IC_50_) was determined based on the relative survival curve.

### ^125^I Uptake Assay

Cells (1.5×10^5^) were seeded in six-well plates, treated with DMSO, AhR antagonist, or transfected with shRNA or plasmid, and incubated for 48 h. Cells were incubated in 1 ml serum-free RPMI 1640 which contains 74 kBq Na^125^I at 37°C for 1 h. The Na^125^I containing medium was withdrawn, and the cells were washed twice in PBS before being lysed on ice with 0.3 M sodium hydroxide. The radioactivity of cell lysates was counted by a *γ* counter. To normalize the cell counts, each group had one well without Na^125^I treatment for counting cell numbers.

### ^131^I Colony Formation Assay

Cells (5 × 10^2^) were seeded into six-well plates, and treated with DMSO, AhR antagonist or transfected with shRNA or plasmid, and incubated for 48 h. Then, the medium was discarded, and cells were washed twice in PBS. One mL of culture medium with or without 20 μCi Na^131^I was added for 6 h, and the radioactive medium was discarded. Cells were cultured in regular medium approximately 1 week. Cell colonies were fixed by 4% paraformaldehyde and stained with crystal violet. The number of cell colonies was determined by counting.

### RNase R and Actinomycin D Treatment, and Quantitative Real-Time PCR (qRT-PCR) Assay

Total RNA was extracted using the RNA-Quick Purification Kit (Vazyme, Shanghai, China). For the RNase R treatment, 2 μg of total RNA was incubated with or without 3 U/mg RNase R (Geneseed, Guangzhou, China) for 15 min at 37°C. For the Actinomycin D treatment, transcription was prevented by the addition of 2 mg/ml Actinomycin D. One μg of total RNA was transcribed into cDNA on an ABI Veriti™ 96-Well Thermal Cycler (Thermo Fisher) using HiScript II Q RT SuperMix for qPCR (R223-01, Vazyme) for mRNA and circRNA analysis, and miRNA 1st Strand cDNA Synthesis Kit (MR101-01, Vazyme) for miRNA analysis. The qRT-PCR reaction was carried out in a Thermofisher 7500 Real-Time PCR system using AceQ Universal SYBR qPCR Master Mix for mRNA and circRNA (Q511-02, Vazyme) and miRNA Universal SYBR qPCR Master Mix (MQ101-01/02, Vazyme). All sequences were presented in Supplementary Table S1.

### Western Blotting Analysis

The cells were lysed in RIPA buffer. Equal amounts of total protein were resolved, transferred to PVDF membranes (Millipore) and immunoblotted with the specified primary antibodies. Membranes were hybridized with the following primary antibodies: AhR (Abcam, #ab190797, 1:1000), NIS (Abcam, #ab242007, 1:1000), IGF2BP2 (Abcam, #ab124930, 1:2000), Histone 3 (Abcam, # ab267372, 1:1000) and β-actin (Abcam, #ab8227, 1:1000). Species-specific HRP-conjugated antibodies (1:5000; Cell Signaling Technology) were used to hybridize membranes. The cytoplasm and nuclear proteins were recovered and separated using the nuclear and Cytoplasmic Protein Extraction Kit (Yeasen, Shanghai, China), following cell lysis. The Potent ECL kit (Yeasen, Shanghai, China) was used to visualize the bands.

### Immunofluorescent Localization of Na/I Symporter

Cells were seeded in six-well chamber slides and treated with StemRegenin 1 (100 nM). After 48 h, the cells were washed twice with PBS and fixed for 15 min with 4% paraformaldehyde. The cells were blocked with 1% BSA for 1 h. The cells were then treated with rabbit anti-NIS (1:100; Abcam), and goat Anti-Rabbit IgG H&L (Alexa Fluor® 647) (Abcam) diluted at 1:100, followed by DAPI.

### Dual-Luciferase Reporter Assay

The wild-type sequences of cicrSH2B3 and IGF2BP2 3′-UTR as well as their corresponding mutant sequences targeting hsa-miR-4640-5p binding sites were synthesized. These sequences were subcloned into the luciferase reporter vector cicrSH2B3 (Genomeditech, Shanghai, China) respectively. Vectors were co-transfected into K1 cells by HG transgene reagent (Genomeditech, Shanghai, China) according to the manufacturer’s instructions. The relative luciferase activities were determined using the Dual-luciferase Assay Kit (Genomeditech, Shanghai, China) following the manufacturer’s instructions.

### Immunohistochemistry (IHC)

For IHC, 5-μm thyroid cancer sections were treated with xylene, 100% alcohol, 95% alcohol, 85% alcohol, 75% alcohol and then Tris/EDTA buffer was used for antigen retrieval. Then the samples were incubated with anti-AhR (1:500 dilution in PBS) for 2 h. After PBS washing, HRP-conjugated secondary (1:500 dilution in PBS) and 3, 3′-diaminobenzidine were used for visualization. All staining was scored using a semiquantitative method in terms of the staining intensity and the positive rate. In detail, the staining intensity was determined as 0 = negative, 1 = weak, 2 = moderate, and 3 = strong, while the positive rate was defined as 0, < 1%; 1, 1–25%; 2, 26–50%; 3, 51–75%; and 4, 75–100%. IHC scoring > 6 and ≤ 6 were defined as high and low expression, respectively (Xu et al., 2019).

### Statistical Analysis

The data is represented as means ± standard deviation. SPSS 22.0 (SPSS, Chicago, IL, United States) was used to perform all the statistical analyses. A one-way analysis of variance was used to assess differences across groups, while the Student’s *t*-test was used to assess differences between groups. The statistical significance level was set at *p* < 0.05.

## Results

### AhR Antagonists Induce Differentiation of PTC

**FIGURE 1:**
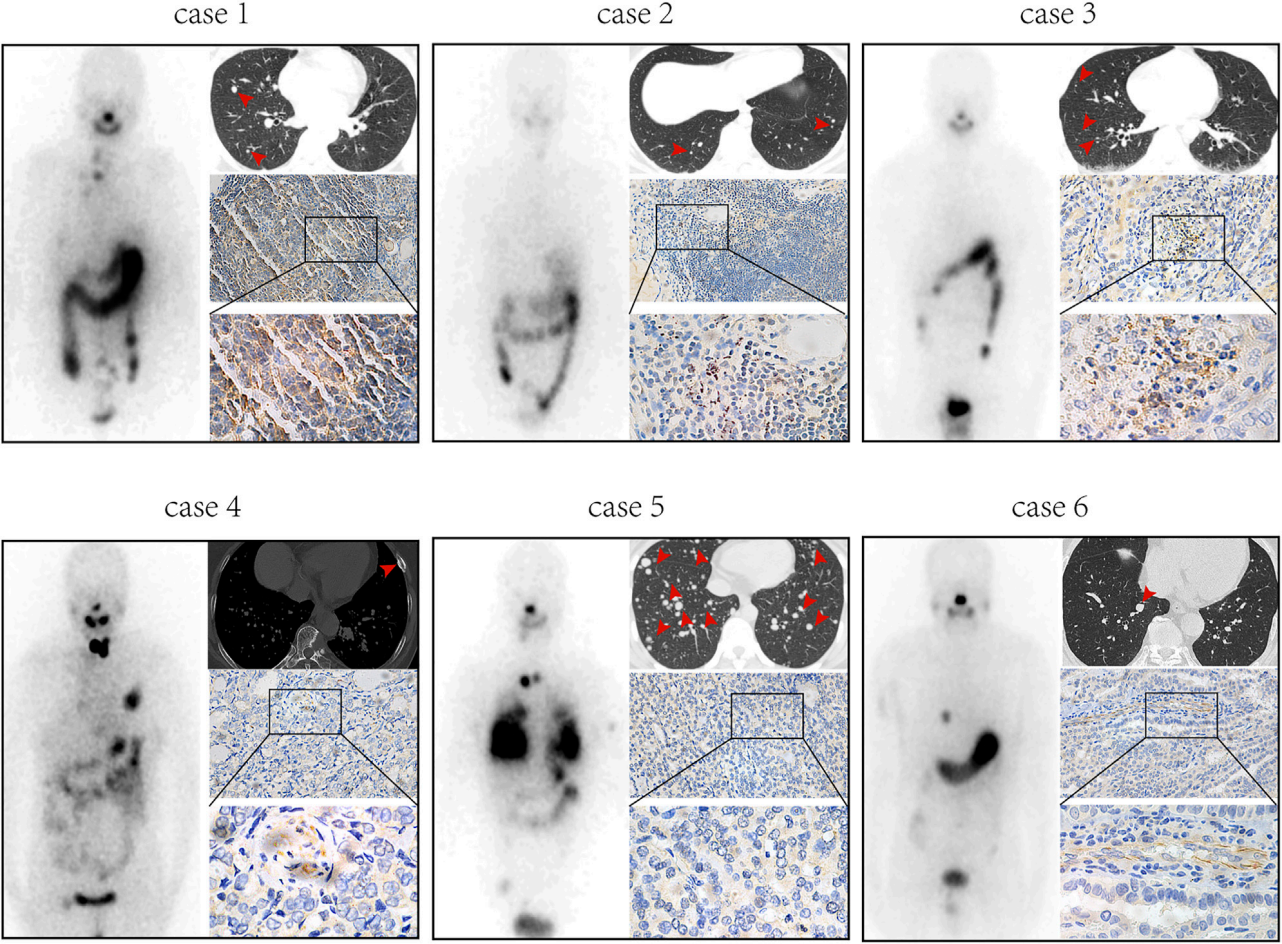
AhR is associated with ^131^I uptake of lesions in PTC patients with lymph node or distant metastases. Post-therapeutic whole body scan, CT, and immunohistochemistry of AhR in patients with high expression of AhR (cases 1-3) and patients with low expression of AhR (cases 4-6).

**FIGURE 2:**
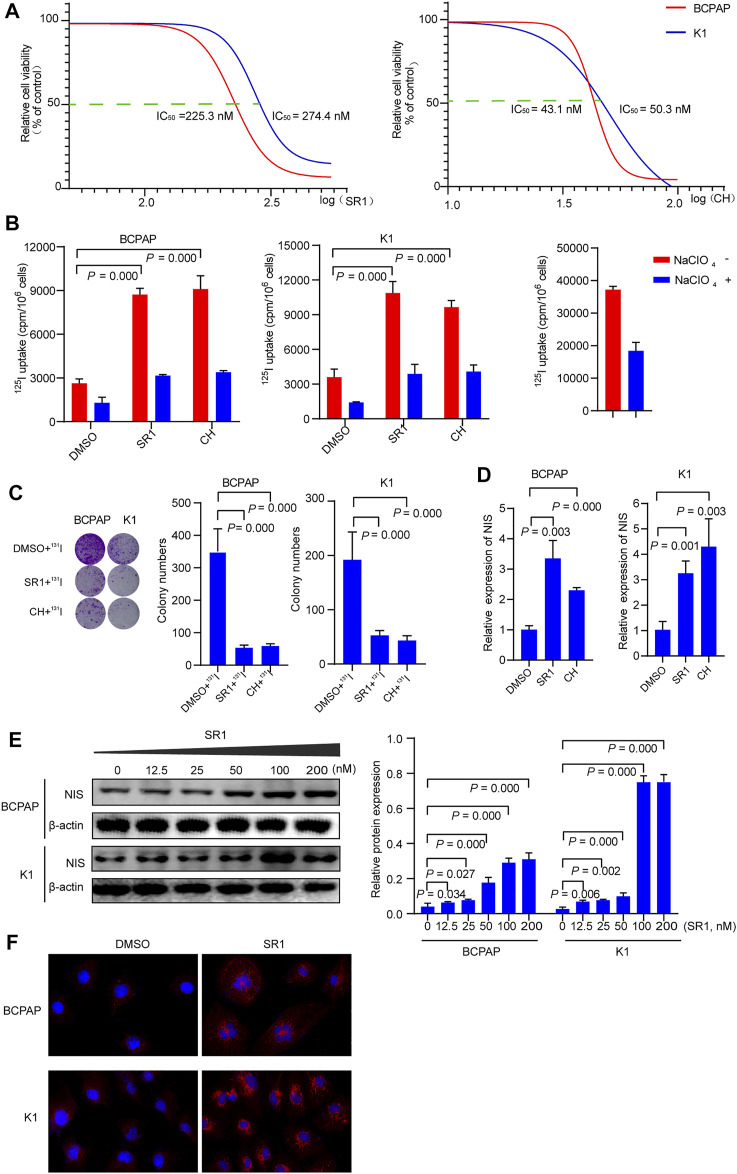
AhR antagonists promote differentiation of PTC cells. **(A)** IC_50_ of SR1 and CH in BCPAP and K1 cells. **(B)** ^125^I uptake in BCPAP and K1 cells treated with SR1 and CH. **(C)** ^131^I colony formation in BCPAP and K1 cells treated with SR1 and CH. **(D)** Relative expression of NIS in BCPAP and K1 cells treated with SR1 and CH. **(E)** The protein levels of NIS in BCPAP and K1 cells treated with SR1. **(F)** Localization of NIS in BCPAP and K1 cells treated with SR1. SR1, StemRegenin 1; CH, CH-223191.

Previously published findings have indicated that AhR was up-regulated in thyroid cancer, but the role of AhR in the differentiation of PTC was still unclear (Moretti et al., 2020). According to AhR levels, clinicopathological data deriving from a total of 32 PTC patients with lymph node or distant metastases were analyzed, in which only 4 (22.2%) patients in high AhR group showed ^131^I positive lesions on post-therapeutic whole body scan (Rx-WBS) after the initial ^131^I treatment, while 10 (71.4%) patients in low AhR group showed ^131^I positive lesions on Rx-WBS after the initial ^131^I treatment. AhR was correlated with condition of ^131^I uptake of lesions after the initial ^131^I treatment (*p* = 0.011) (Table 1), indicating that AhR might play an important role in the differentiation of PTC. Representative cases were shown in Figure 1. Further, we used AhR antagonists, StemRegenin 1 (SR1) and CH-223191 (CH) in PTC cells (BCPAP and K1) to evaluate the differentiation of PTC. The IC_50_ value for SR1 and CH were 225.3 and 43.1 nM in BCPAP, while 274.4 and 50.3 nM in K1, respectively (Figure 2A). SR1 or CH was administered to stimulate the differentiation of PTC cells for 48 h. ^125^I uptake of BCPAP cells treated with SR1 (100 nM) or CH (20 nM) was 3.32-, and 3.46-fold higher than those of control cells (both *p* < 0.05), and 3.02- and 2.67-fold higher in K1 cells (both *p* < 0.05) (Figure 2B). ^131^I colony formation assay showed that the cell colonies in PTC cells treated with SR1 (100 nM) or CH (20 nM) were reduced (Figure 2C). Since NIS is the critical factor which is responsible for differentiation of PTC, the mRNA and protein levels of NIS in PTC cells treated with AhR antagonists are determined. The mRNA expression of NIS was distinctly enhanced in PTC cells treated with SR1 (100 nM) or CH (20 nM) (Figure 2D). With regard to the protein level, various concentration of SR1 (0, 12.5, 25, 50, 100, 200 nM) were used. The protein levels of NIS were significantly increased in PTC cells treated with 50 or 100 nM SR1 (Figure 2E). Furthermore, NIS protein was found to be distinctly localized on the membrane of PTC cells after treated with SR1 (100 nM) (Figure 2F). These results suggested that AhR antagonists promote the differentiation of PTC cells.

**TABLE 1:** Clinicopathological features of patients with metastatic papillary thyroid cancer according to the expression of aryl hydrocarbon receptor (*N* = 32).

Characteristic	High (*n* = 18)	Low (*n* = 14)	*p* value
Age	—	—	0.928
≥55 years	8	6	—
<55 years	10	8	—
Sex	—	—	0.957
Female	14	11	—
Male	4	3	—
Size	—	—	0.301
≤2.0 cm	9	10	—
2.0 ∼ 4.0 cm	5	2	—
>4.0 cm	4	2	—
Metastatic site(s)	—	—	0.667
Lymph node-only	6	4	—
Lung-only	4	3	—
Bone-only	2	1	—
Others or combined	6	6	—
Rx-WBS after initial ^131^I therapy	—	—	0.011^a^
^131^I-avid	4	10	—
Non-^131^I-avid	14	4	—
Tg_on_ (ng/ml)	53.7 ± 13.2	48.9 ± 23.5	0.338
Tg_off_ (ng/ml)	374.7 ± 79.1	294.2 ± 109.3	0.673

^131^I, radioiodine; Rx-WBS, post-therapeutic whole body scan; Tg_on_, suppressed thyroglobulin; Tg_off_, stimulated thyroglobulin.

a Statistical significant difference.

### Differentiation Associated hsa_circ_0006741 is Down-regulated in AhR Antagonist Treated PTC Cells

**FIGURE 3:**
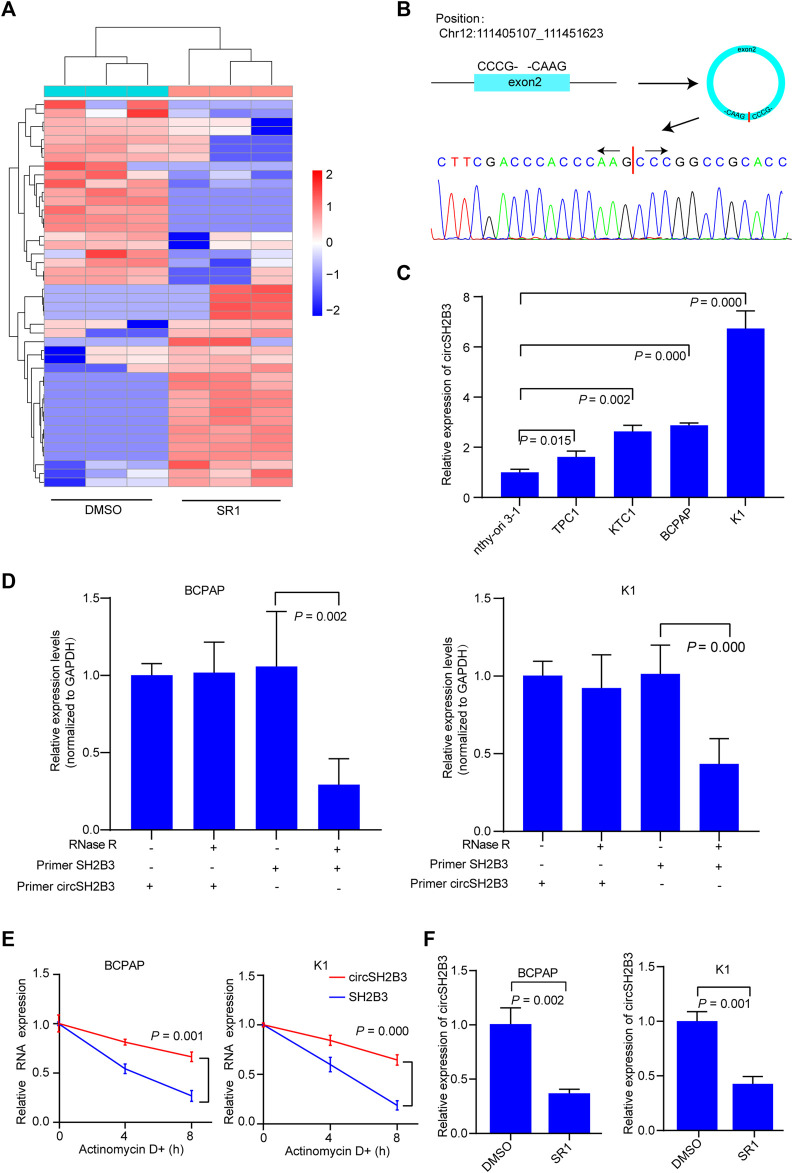
CircSH2B3 is down-regulated in SR1 treated PTC cells. **(A)** Heatmap of different expressed circRNAs in SR1 treated K1 cells and control cells. Red indicated up-regulated while green indicated down-regulated. **(B)** Schematic illustration demonstrating SH2B3 exon 2 circularization to form circSH2B3. The presence of circSH2B3 is validated by Sanger sequencing. **(C)** Relative expression of circSH2B3 in PTC cell lines. **(D)** Relative expression of circSH2B3 and SH2B3 mRNA in BCPAP and K1 cells treated with or without RNase R. **(E)** Relative expression of circSH2B3 and SH2B3 mRNA in BCPAP and K1 cells treated with Actinomycin D. **(F)** Relative expression of circSH2B3 in BCPAP and K1 cells treated with SR1. SR1, StemRegenin 1.

Using circRNA microarray, we examined the differentially expressed circRNAs in K1 control and SR1 treated K1 cells to determine the potential circRNAs that contributed to the differentiation effect of AhR antagonists. By comparing SR1 treated K1 cells to control cells, we found 23 circRNAs with significant up-regulation and 21 circRNAs with significant down-regulation. Fold change filtering was used to identify differential expression patterns of circRNAs, and a heatmap of these circRNAs was generated (Figure 3A). Among these differentially expressed circRNAs, hsa_circ_0006741 was associated with cell differentiation and was down-regulated in AhR antagonist treated K1 cells. Therefore, we selected hsa_circ_0006741 for further analysis.

Hsa_circ_0006741 is formed by the circularization of exons 2 of the SH2B3 gene with 759 spliced lengths (hereafter referred to as circSH2B3) and located at chr12:111405107_111451623. Sanger sequencing was conducted to confirm the predicted head-to-tail splicing junction in the qRT-PCR product of circSH2B3 identified by its expected size using well-designed divergent primers (Figure 3B). The circSH2B3 expression was higher in multiple PTC cells than in normal thyroid cells (Nthy-ori 3-1), and BCPAP and K1 cells were chosen for further investigation (Figure 3C). Following that, RNase R was applied to treat PTC cells to rule out the possibility that head-to-tail splicing may be produced by genomic rearrangement or trans-splicing. In the RNase R treatment assay, we found that circSH2B3 was resistant to RNase after digestion of RNA (Figure 3D). Also, under the treatment of Actinomycin D, the level of linear SH2B3 was decreased rapidly than that of circSH2B3 over time (Figure 3E). qRT-PCR was used to validate the relative expression of circSH2B3 in SR1 treated BCPAP and K1 cells (Figure 3F). Taken together, these findings suggested that circSH2B3 might be a potential target for SR1.

### CircSH2B3 Plays a Key Role in the Dedifferentiation of PTC

**FIGURE 4:**
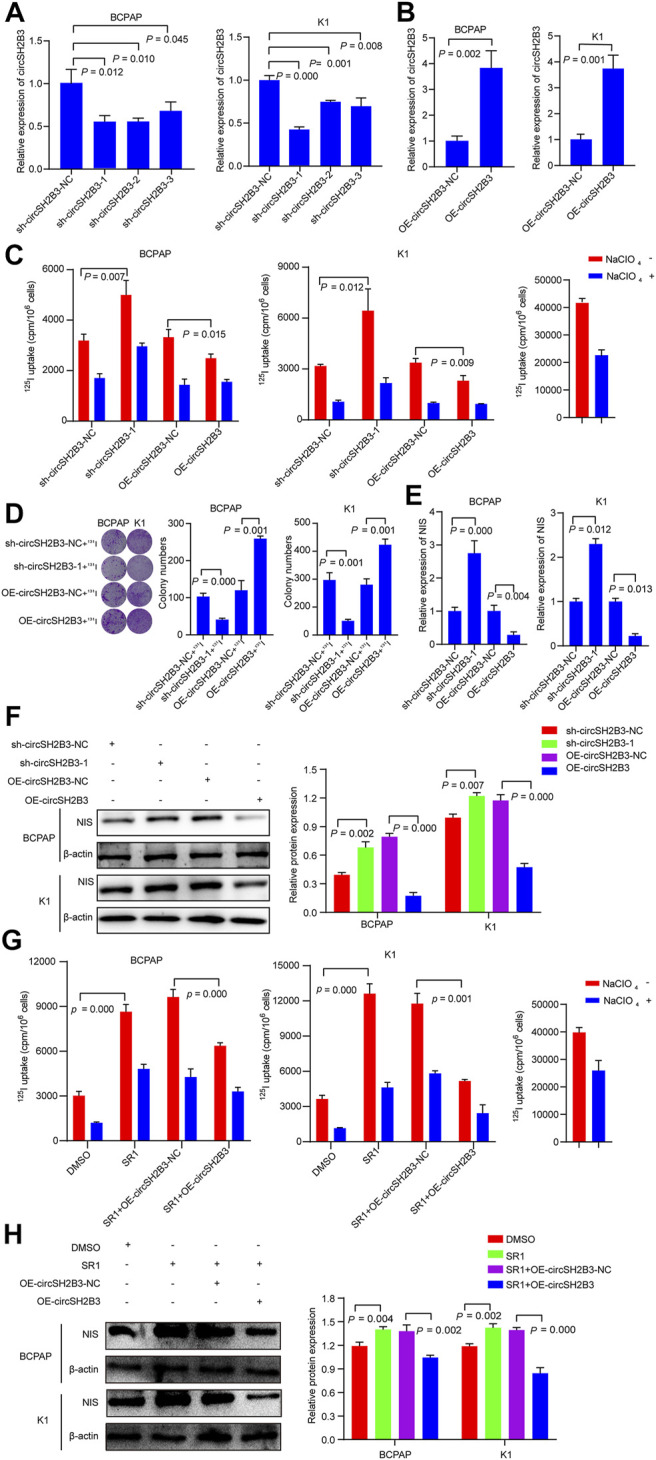
CircSH2B3 plays a key role in SR1 induced differentiation of PTC. **(A,B)** Relative expression of circSH2B3 in BCPAP and K1 cells transfected with circSH2B3 shRNA (sh-circSH2B3) and overexpression (OE-circSH2B3) plasmids. **(C)** ^125^I uptake in BCPAP and K1 cells transfected with sh-circSH2B3 or OE-circSH2B3. **(D)** ^131^I colony formation in BCPAP and K1 cells transfected with sh-circSH2B3 or OE-circSH2B3. **(E)** Relative expression of NIS in BCPAP and K1 cells transfected with sh-circSH2B3 or OE-circSH2B3. **(F)** The protein levels of NIS in BCPAP and K1 cells transfected with sh-circSH2B3 or OE-circSH2B3. **(G)** ^125^I uptake in SR1 treated BCPAP and K1 cells co-transfected with OE-circSH2B3-NC or OE-circSH2B3. **(H)** The protein levels of NIS in SR1 treated BCPAP and K1 cells co-transfected with OE-circSH2B3-NC or OE-circSH2B3. SR1, StemRegenin 1.

To further investigated the dedifferentiation of circSH2B3 in PTC cells, the circSH2B3 silencing (sh-cicrSH2B3) and overexpression (OE-circSH2B3) were constructed using the plasmids transfection (Figures 4A,B). We then performed ^125^I uptake assay and ^131^I colony formation assay to determine the dedifferentiation effect of circSH2B3. The ^125^I uptake assay revealed that when the expression of circSH2B3 was inhibited, the ^125^I uptake was increased, while when the expression of circSH2B was enhanced, the ^125^I uptake was significantly reduced as compared to control groups (Figure 4C). Similarly, colony formation in circSH2B3 knockdown cells was inhibited but enhanced in circSH2B3 overexpression cells (Figure 4D). In addition, mRNA and protein levels of NIS were increased in sh-circSH2B3 PTC cells but down-regulated in OE-circSH2B3 PTC cells (Figures 4E,F). We also investigated whether acquisition of circSH2B3 could affect the differentiation of SR1, the circSH2B3 was overexpressed in SR1 treated PTC cells. The increase of cirSH2B3 expression decreased ^125^I uptake and the protein levels of NIS in SR1 treated PTC cells (Figures 4G,H). These results indicated that SR1 promotes differentiation of PTC cells via inhibiting circSH2B3.

### CircSH2B3 Acts as a Sponge for hsa-miR-4640-5p

**FIGURE 5:**
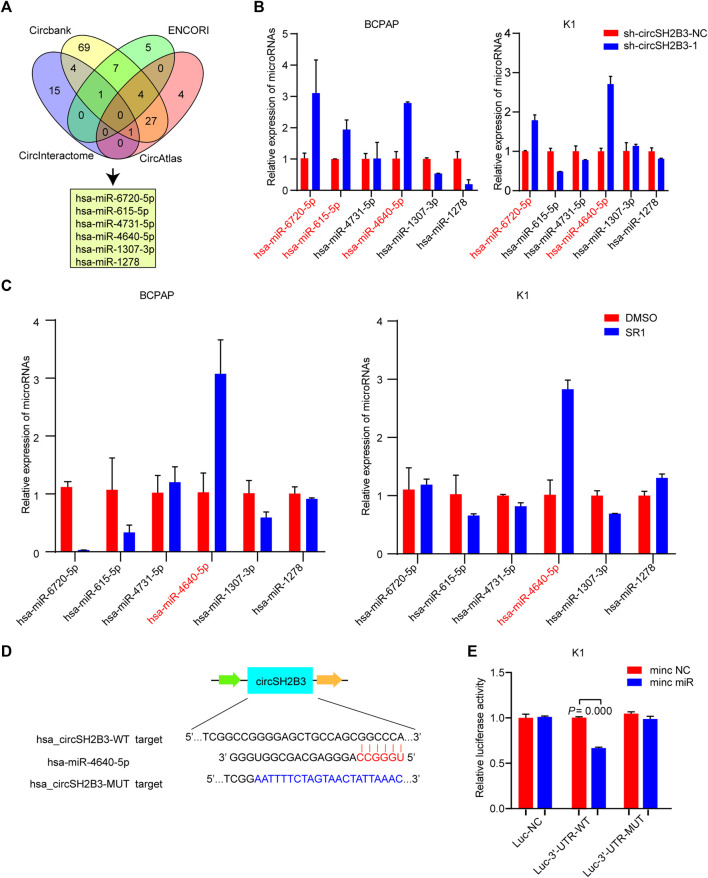
circSH2B3 acts as a sponge for hsa-miR-4640-5p in PTC cells. **(A)** Venn diagram of hsa-miR-6720-5p, hsa-miR-615-5p, hsa-miR-4731-5p, hsa-miR-4640-5p, hsa-miR-1307-3p and hsa-miR-1278. **(B)** Relative miRNA expression in BCPAP and K1 cells transfected with sh-circSH2B3. **(C)** Relative miRNA expression in BCPAP and K1 cells treated with SR1. **(D)** The bioinformation analysis showing the binding site of circSH2B3 and hsa-miR-4640-5p. **(E)** The luciferase activity assay showing the combination of circSH2B3 and hsa-miR-4640-5p. SR1, StemRegenin 1.

One of the main biological roles of circRNAs is to sponge to miRNA and affect the mRNA targeted by the corresponding miRNA (Meng et al., 2017). To investigate the underlying molecular mechanisms of circSH2B3 in dedifferentiation of PTC, we screened the target miRNAs of circSH2B3 via bioinformatics analysis. Circbank (http://www.circbank.cn), circAtlas (http://circatlas.biols.ac.cn), CircInteractome (https://circinteractome.nia.nih.gov/) and ENCORI (http://starbase.sysu.edu.cn/), were used to predict targets miRNAs of circSH2B3. The overlap across the databases revealed a total of 6 miRNAs (hsa-miR-6720-5p, hsa-miR-615-5p, hsa-miR-4731-5p, hsa-miR-4640-5p, hsa-miR-1307-3p and hsa-miR-1278) (Figure 5A). The expression of these miRNAs in circSH2B3 knockdown PTC cells was then confirmed. Hsa-miR-6720-5p and hsa-miR-4640-5p were increased in PTC cells transfected with sh-circSH2B3 (Figure 5B). Furthermore, we selected the hsa-miR-4640-5p as the target miRNA via comparing miRNA expression in PTC cells treated with SR1 to control cells (Figure 5C). To confirm the interaction between circSH2B3 and hsa-miR4640-5p, we constructed dual-luciferase reporter vectors containing wild (WT) and mutant (Mut) circSH2B3 sequences capable of binding hsa-miR-4640-5p (Figure 5D). After transfecting vectors into K1 cells with or without a hsa-miR-4640-5p mimic and circSH2B3, dual-luciferase assays revealed that hsa-miR-4640-5p suppressed luciferase activity in WT cells but not in Mut cells (Figure 5E). These results indicated that hsa-miR-4640-5p is a direct downstream target of circSH2B3, involving in cell differentiation of PTC.

### Hsa-miR-4640-5p Silencing Reverses the Differentiation Effect of sh-circSH2B3

**FIGURE 6:**
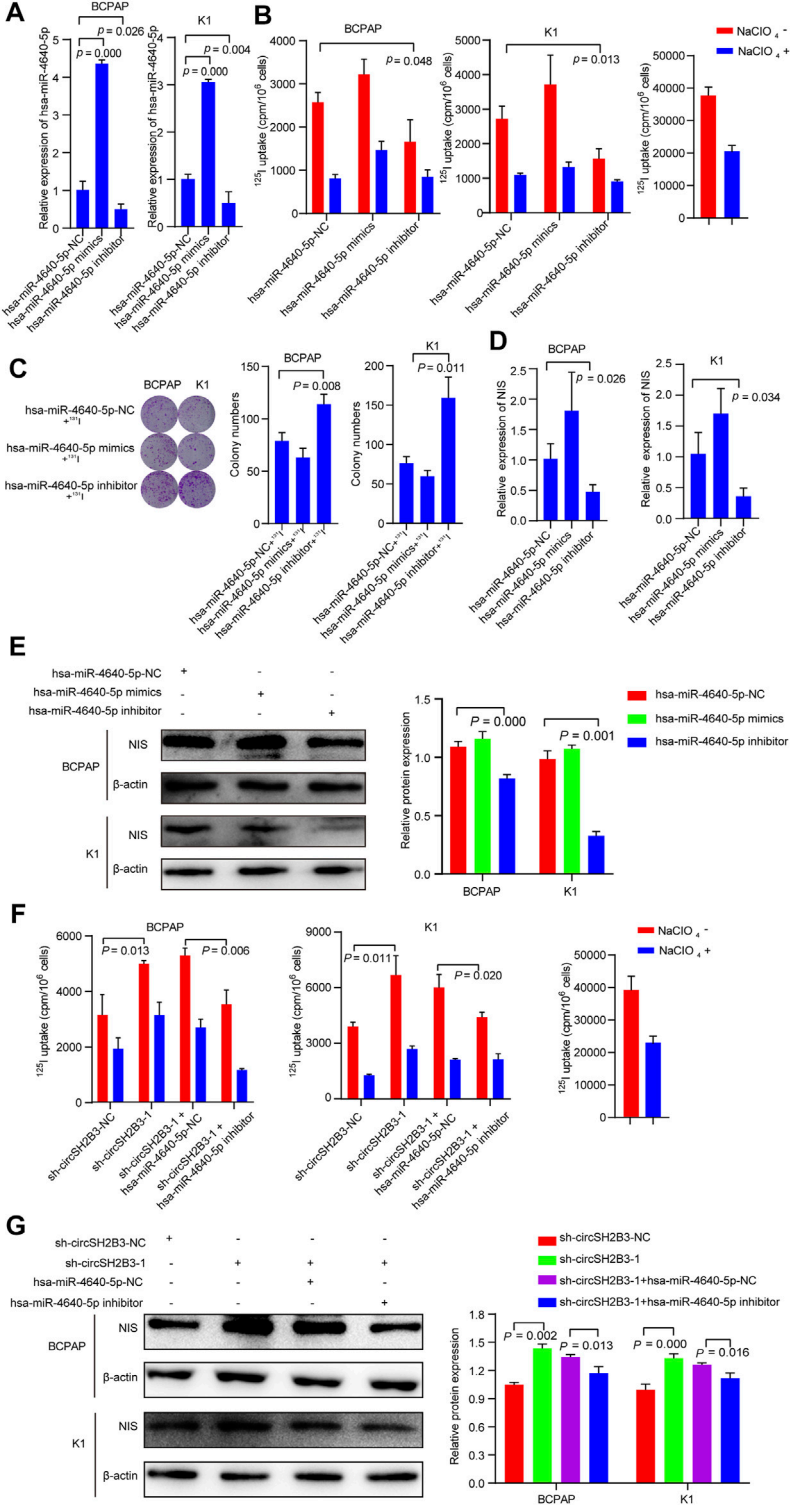
hsa**-**miR-4640-5p silencing reverses the sh-circSH2B3 induced differentiation of PTC cells. **(A)** Relative expression of hsa-miR-4640-5p in BCPAP and K1 cells transfected with hsa-miR-4640-5p mimics or inhibitor. **(B)** ^125^I uptake in BCPAP and K1 cells transfected with hsa-miR-4640-5p mimics or inhibitor. **(C)** ^131^I colony formation in BCPAP and K1 cells transfected with hsa-miR-4640-5p mimics or inhibitor. **(D)** Relative expression of NIS in BCPAP and K1 cells transfected with hsa-miR-4640-5p mimics or inhibitor. **(E)** The protein levels of NIS in BCPAP and K1 cells transfected with hsa-miR-4640-5p mimics or inhibitor. **(F)** ^125^I uptake in sh-circSH2B3 BCPAP and K1 cells co-transfected with inhibitor NC or hsa-miR-4640-5p inhibitor. **(G)** The protein levels of NIS in sh-circSH2B3 BCPAP and K1 cells co-transfected with inhibitor NC or hsa-miR-4640-5p inhibitor.

To determine the differentiation effect of hsa-miR-4640-5p in PTC cells, we transfected BCPAP and K1 cells with the hsa-miR-4640-5p mimics or inhibitor and the transfection efficiency was detected by qRT-PCR (Figure 6A). Hsa-miR-4640-5p inhibitor decreased the ^125^I uptake in PTC cells, while hsa-miR-4640-5p mimics only slightly increased ^125^I uptake in both BCPAP and K1 cells (Figure 6B). The ^131^I colony formation assay showed increased colony numbers in PTC cells transfected with hsa-miR-4640-5p inhibitor, and slightly decreased colony numbers in PTC cells transfected with hsa-miR-4640-5p mimics (Figure 6C). Furthermore, the qRT-PCR and western blot were performed to evaluate the relative mRNA and protein levels of NIS in PTC cells transfected wih hsa-miR-4640-5p mimics or inhibitor. As shown in Figures 6D,E, the relative mRNA and protein levels of NIS in PTC cells were reduced following transfection with hsa-miR-4640-5p inhibitors but increased slightly after transfection with hsa-miR-4640-5p mimics. These results suggested that hsa-miR-4640-5p inhibitors act as suppressors in differentiation of PTC.

As we hypothesized that silencing circSH2B3 promotes differentiation of PTC primarily by sponging hsa-miR-4640-5p, it was necessary to determine whether a hsa-miR-4640-5p inhibitor might reverse the sh-circSH2B3 induced differentiation of PTC cells. Rescue experiments were performed by co-transfecting sh-circSH2B3-NC or sh-circSH2B3 in PTC cells with a hsa-miR-4640-5p inhibitor or its control. As shown by the ^125^I uptake assay, the hsa-miR-4640-5p inhibitor partially weakened the sh-circSH2B3 induced differentiation effect of PTC cells (Figure 6F), as well as protein levels of NIS (Figure 6G). These results suggested that hsa-miR-4640-5p slightly promotes differentiation of PTC, and the hsa-miR-4640-5p suppression partially inhibited the sh-circSH2B3 induced differentiation of PTC.

### IGF2BP2 is a Direct Target of Hsa-miR-4640-5p

**FIGURE 7:**
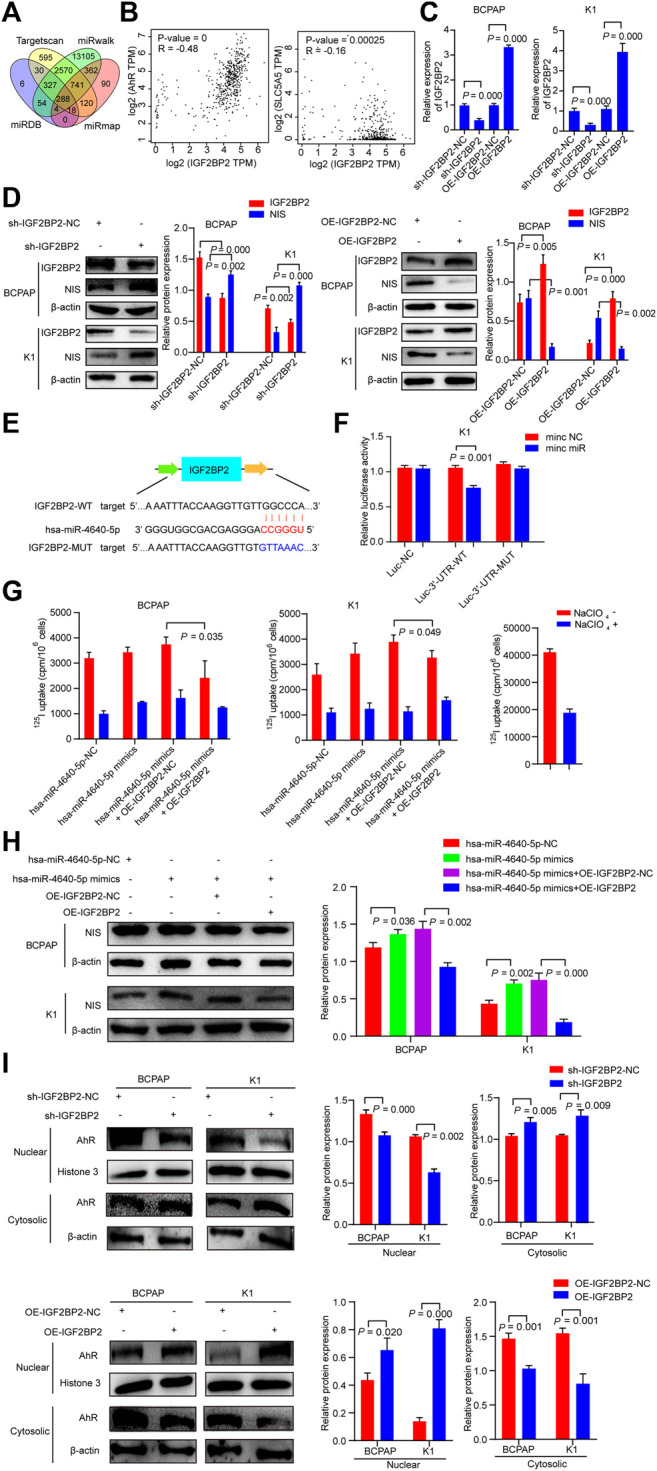
IGF2BP2 is a direct target of hsa-miR-4640-5p. **(A)** Bioinformatic analysis of the potential target gene by prediction software (miRmap, miRwalk, Targetscan, miRDB). **(B)** Pearson analyses showing the correlation between IGF2BP2 and AhR, IGF2BP2 and NIS based on GEPIA (http://gepia.cancer-pku.cn/index.html) databases. **(C)** Relative expression of IGF2PB2 in BCPAP and K1cells transfected with IGF2BP2 shRNA (sh-IGF2BP2) and overexpression (OE-IGF2BP2) plasmids. **(D)** The protein levels of NIS and IGF2BP2 in BCPAP and K1cells transfected with sh-IGF2BP2 or OE-IGF2BP2. **(E)** The bioinformation analysis showing the binding site of hsa-miR-4640-5p and IGF2BP2. **(F)** The luciferase activity assay showing the combination of hsa-miR-4640-5p and IGF2BP2. **(G)** ^125^I uptake in hsa-miR-4640-5p mimics treated BCPAP and K1 cells co-transfected with OE-IGF2BP2-NC or OE-IGF2BP2. **(H)** The protein levels of NIS in hsa-miR-4640-5p mimics treated BCPAP and K1 cells co-transfected with OE-IGF2BP2-NC or OE-IGF2BP2. **(I)** The protein levels of AhR in nuclear and cytosolic in BCPAP and K1 cells transfected with sh-IGF2BP2 or OE-IGF2BP2.

The target genes with the potentiality of hsa-miR-4640-5p were picked out by the prediction software (miRmap, miRwalk, Targetscan, miRDB). Based on the bioinformatic analysis, the potential target gene was identified as Insulin-like growth factor 2 (IGF2) mRNA-binding protein 2 (IGF2BP2) (Figure 7A). According to the GEPIA (http://gepia.cancer-pku.cn/index.html) databases, the Pearson analysis revealed that IGF2BP2 was positively associated with AhR, and negatively correlated with NIS (Figure 7B). Stably suppressed and overexpressed IGF2BP2 (sh-IGF2BP2 and OE-IGF2BP2) were constructed by lentivirus transfection (Figure 7C). Western blot was performed to validate the involvement of IGF2BP2 in the dedifferentiation of PTC, which revealed that the protein levels of NIS were up-regulated in stably knockdown IGF2BP2 PTC cells, but down-regulated in stably overexpression IGF2BP2 PTC cells (Figure 7D). Following the discovery of the binding site of hsa-miR-4640-5p and IGF2BP2 through bioinformatics analysis, the luciferase WT and Mut type plasmids were constructed based on the binding site (Figure 7E). The luciferase activity assay was conducted to address the interaction between hsa-miR-4640-5p and IGF2BP2, revealing that hsa-miR-4640-5p binds to the IGF2BP2 3′-UTR (Figure 7F). In addition, ^125^I uptake assay and western blot analyses revealed that the slightly enhanced differentiation effect of hsa-miR-4640-5p could be obviously inhibited by IGF2BP2 overexpression (Figures 7G,H).

Cytoplasmic AhR translocates to the nucleus, and heterodimerizes with aryl hydrocarbon receptor nuclear translocator (ARNT), and mediates a variety of biological and toxicological effects by inducing the transcription of AhR-responsive genes. IGF2BP2, as an N6-methyladenosine (m6A) reader, it might participate in AhR translocation to promote the function of AhR. Thus, we extracted the protein of the nucleus and cytoplasm of sh-IGF2BP2 or OE-IGF2BP2 PTC cells. The results showed that nucleus AhR protein levels in the sh-IGF2BP2 group were down-regulated, while that in the OE-IGF2BP2 group was up-regulated compared to the control groups (Figure 7I). These data above indicated that IGF2BP2 is a direct target of hsa-miR-4640-5p, involving in the dedifferentiation of PTC, as well as activation of AhR.

### sh-circSH2B3 and SR1 Promote Differentiation of PTC by Inhibiting IGF2BP2

**FIGURE 8:**
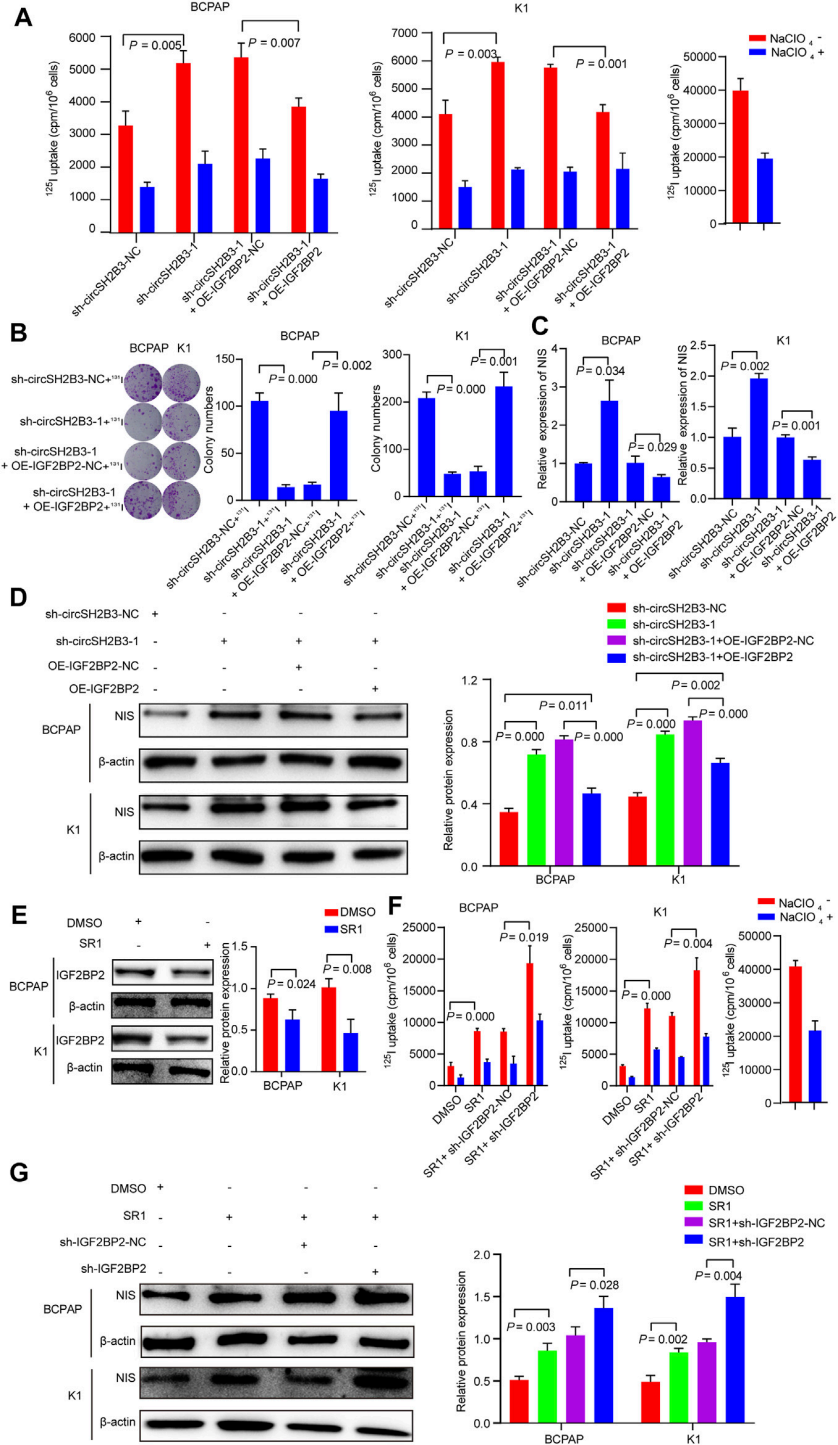
sh-circSH2B3 and SR1 promote differentiation of PTC via targeting IGF2BP2. **(A)** ^125^I uptake in sh-circSH2B3 BCPAP and K1 cells co-transfected with OE-IGF2BP2-NC or OE-IGF2BP2. **(B)** ^131^I colony formation in sh-circSH2B3 BCPAP and K1 cells co-transfected with OE-IGF2BP2-NC or OE-IGF2BP2. **(C)** Relative expression of NIS in sh-circSH2B3 BCPAP and K1 cells co-transfected with OE-IGF2BP2-NC or OE-IGF2BP2. **(D)** The protein levels of NIS in sh-circSH2B3 BCPAP and K1 cells co-transfected with OE-IGF2BP2-NC or OE-IGF2BP2. **(E)** The protein levels of IGF2BP2 in BCPAP and K1 cells treated with SR1. **(F)** ^125^I uptake in SR1 treated BCPAP and K1 cells co-transfected with sh-IGF2BP2-NC or sh-IGF2BP2. **(G)** The protein levels of NIS in SR1 treated BCPAP and K1 cells co-transfected with sh-IGF2BP2-NC or sh-IGF2BP2. SR1, StemRegenin 1.

To investigate whether sh-circSH2B3 promoted PTC differentiation by targeting IGF2BP2, the impact of IGF2BP2 overexpression on circSH2B3-deficient PTC cells was assessed using the ^125^I uptake assay (Figure 8A) and ^131^I colony formation assay (Figure 8B), which demonstrated that high levels of IGF2BP2 could restrict the differentiation effect of circSH2B3-deficient cells. The relative mRNA and protein levels of NIS were also examined in sh-circSH2B3 PTC cells co-transfected with OE-IGF2BP2-NC or OE-IGF2BP2. The relative mRNA and protein levels of NIS increased in sh-circSH2B3 PTC cells but decreased in sh-circSH2B3 PTC cells co-transfected with OE-IGF2BP2 (Figures 8C,D). These findings suggested that IGF2BP2 inhibits the sh-circSH2B3 induced differentiation of PTC.

Considering our previous findings indicated that SR1 results in differentiation of PTC cells via inhibiting circSH2B3, and inhibiting circSH2B3 facilitates differentiation of PTC cells via regulating miR-4640-5p/IGF2BP2 axis, we hypothesized that IGF2BP2 might be a target of SR1. As we expected, the use of SR1 (100 nM) reduced the protein levels of IGF2BP2 (Figure 8E). We further hypothesized that suppressing IGF2BP2 would synergize SR1 to obtain a robust differentiation of PTC, therefore, PTC cells were treated with SR1 (100 nM) and transfected with sh-IGF2BP2. The ^125^I uptake assay and the protein levels of NIS revealed that targeting IGF2BP2 enhanced the differentiation effect of SR1 in PTC cells, as anticipated (Figures 8F,G). These findings imply that SR1 induces differentiation of PTC by targeting IGF2BP2.

## Discussion

**FIGURE 9:**
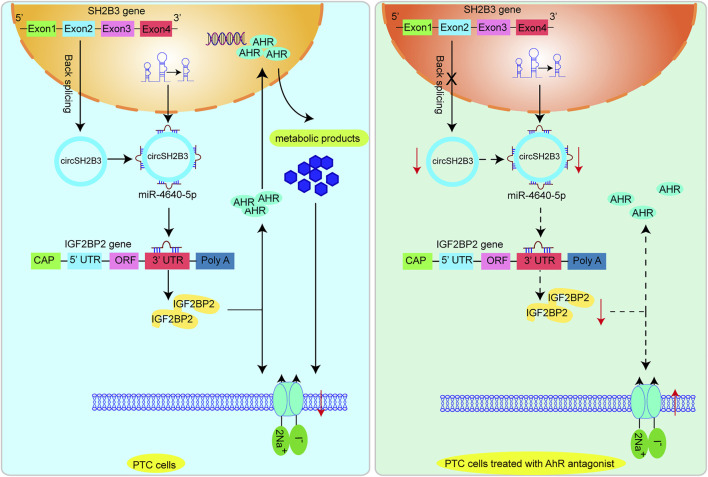
A proposed model that AhR antagonist promotes differentiation of PTC *via* regulating circSH2B3/ miR-4640-5p/IGF2BP2 axis.

Strategies for increasing NIS expression and restoring ^131^I are of critical for RR-PTC management (Ravera et al., 2017). In this work, we initially confirmed that AhR level was associated with differentiation level of PTC and AhR antagonists promoted differentiation of PTC. Mechanistically, the AhR antagonists suppressed the activity of circSH2B3/miR-4640-5p/IGF2BP2 axis to enhance the differentiation of PTC, which may be of great value for providing new targets for the differentiation therapy of PTC. Figure 9 shows the functioning model.

AhR antagonists prevent the receptor from translocating into the nucleus, where it releases the chaperone complex and heterodimerizes with the ARNT. AhR antagonists have been proven to be antioncogenic roles in several cancers, including lung, head and neck, and oral cancers etc. (DiNatale et al., 2011; DiNatale et al., 2012; Kolluri et al., 2017). Previous research found that AhR was overexpressed in thyroid cancer, particularly in BRAF^*V600E*^ mutant ones, and it participated in thyroid cancer initiation and progression by regulating the establishment of an immunosuppressive tumor microenvironment and the epithelial to mesenchymal transition (Moretti et al., 2020). BRAF^*V600E*^-mediated MAPK/ERK signaling may up-regulate AhR expression, thus enhancing CYP2S1 transcription, whereas CYP2S1 in turn enhances transcriptional activity of AhR through its metabolites. This AhR/CYP2S1 feedback loop strongly amplifies oncogenic role of BRAF^*V600E*^ in thyroid cancer cells (Li et al., 2020). The high expression of AhR in thyroid cancer, as well as its oncogenic function in thyroid cancer, prompted us to investigate whether inhibiting AhR nuclear translocation by AhR antagonists induces differentiation of PTC. As anticipated, AhR level was closely associated with the ^131^I uptake of lesions. Furthermore, inhibiting AhR activity with particular antagonists offered a possible direction of differentiation therapy in PTC cells, as shown by increased ^125^I uptake and NIS expression. Although our findings were obtained *in vitro*, we propose AhR-impairment as a novel approach for inducing differentiation of PTC.

The biological process of AhR is explained by a variety of underlying processes. In the present study, we focused on the differentially expressed circRNAs that contributed to cell differentiation to investigate the AhR antagonist induced differentiation of PTC. CirRNAs are a novel class of endogenous RNAs that are generated by non-canonical back-splicing events, exerting a role in gene transcription and translation (Lei et al., 2020). Several circRNAs, such as circRUNX1 (Chu et al., 2021), circDOCK1 (Cui and Xue, 2020), circFNDC3B (Wu et al., 2020) etc. are implecated in PTC progression. In this study, we discovered that the down-regulated circRNA—circSH2B3, derived from SH2B3, is the primary contributor to the AhR antagonist induced differentiation of PTC.

CircRNAs execute their biological function by competitively reducing the account of active miRNA in a ceRNA-dependent manner (Kristensen et al., 2019). miRNAs are a large family of posttranscriptional regulators of gene expression that regulate numerous developmental and cellular processes in eukaryotic organisms (Krol et al., 2010). In this study, hsa-miR-4640-5p exhibited a high binding capacity with circSH2B3, which was validated by qRT-PCR and luciferase, and was found to play a significant role in AhR antagonist induced differentiation. Previously, hsa-miR-4640-5p was shown to directly bind to the 3′-UTR region of eIF5A mRNA, inhibiting the expression of eIF5A in non-small cell lung cancer (Liu et al., 2021). In our study, IGF2BP2 was identified as a target gene of hsa-miR-4640-5p via bioinformatics analysis, qRT-PCR and luciferase. Accumulating evidence highlights the significance of IGF2BP2 as a key regulator of cancer.

IGF2BP2 is an m6A reader, serving as a posttranscriptional regulatory factor (Hu et al., 2020). It predominantly localizes in the cytoplasm, where it can bind to the target mRNAs to enhance their mRNA stability (Bell et al., 2013), and mediate their translation spatially and temporally (Cao et al., 2018). Based on the TGCA database, IGF2BP2 expression was notably up-regulated in thyroid cancer relative to normal tissues (Wang et al., 2020). IGF2BP2 knockdown suppressed cell proliferation, migration, invasion, and induced cell apoptosis of thyroid cancer *via* by reducing the expression of long non-coding RNA HAGLR (Dong et al., 2021). In this study, differentiation of PTC was enhanced after IGF2BP2 knockdown which was shown as increase of ^125^I uptake and NIS expression. Conversely, IGF2BP2 overexpression hampered the differentiation of PTC. Importantly, IGF2BP2 knockdown synergized the differentiation effect of AhR antagonist. Additionally, controlled by the nuclear export signals in the KH domains, IGF2BP2 can transport target transcripts out of the nucleus (Nielsen et al., 2003). We discovered IGF2BP2 suppression decreased the amount of AhR protein levels in nucleus, whereas, IGF2BP2 overexpression increased AhR protein levels in nucleus. The nuclear translocation of AhR in response to ligands may be directly linked to transcriptional activation of target genes. The released metabolic products after AhR activation may further influence the differentiation of PTC, which needs research in the future study.

## Conclusion

Our findings firstly demonstrated that AhR antagonist can promote the differentiation of PTC *via* inhibiting differentiation associated circRNA circSH2B3. As a sponge of hsa-miR-4640-5p, circSH2B3 could up-regulate expression of IGF2BP2, and induce dedifferentiation of PTC. Collectively, our findings provide a new treatment strategy for differentiation of PTC by AhR antagonist, and circSH2B3/miR-4640-5p/IGF2BP2 network may be a potential target for differentiation therapy of PTC and deserves further clinical research.

## Data Availability

The datasets presented in this study can be found in online repositories. The names of the repository/repositories and accession number(s) can be found below: https://www.ncbi.nlm.nih.gov/geo/, accession ID: GSE188657.
