# Supplementary figures and images for: AhR Antagonist Promotes Differentiation of Papillary Thyroid Cancer via Regulating circSH2B3/miR-4640-5P/IGF2BP2 Axis
Source: Front Pharmacol. 2021 Dec 23;12:795386. doi: 10.3389/fphar.2021.795386 (PMC8733664; doi:10.3389/fphar.2021.795386)

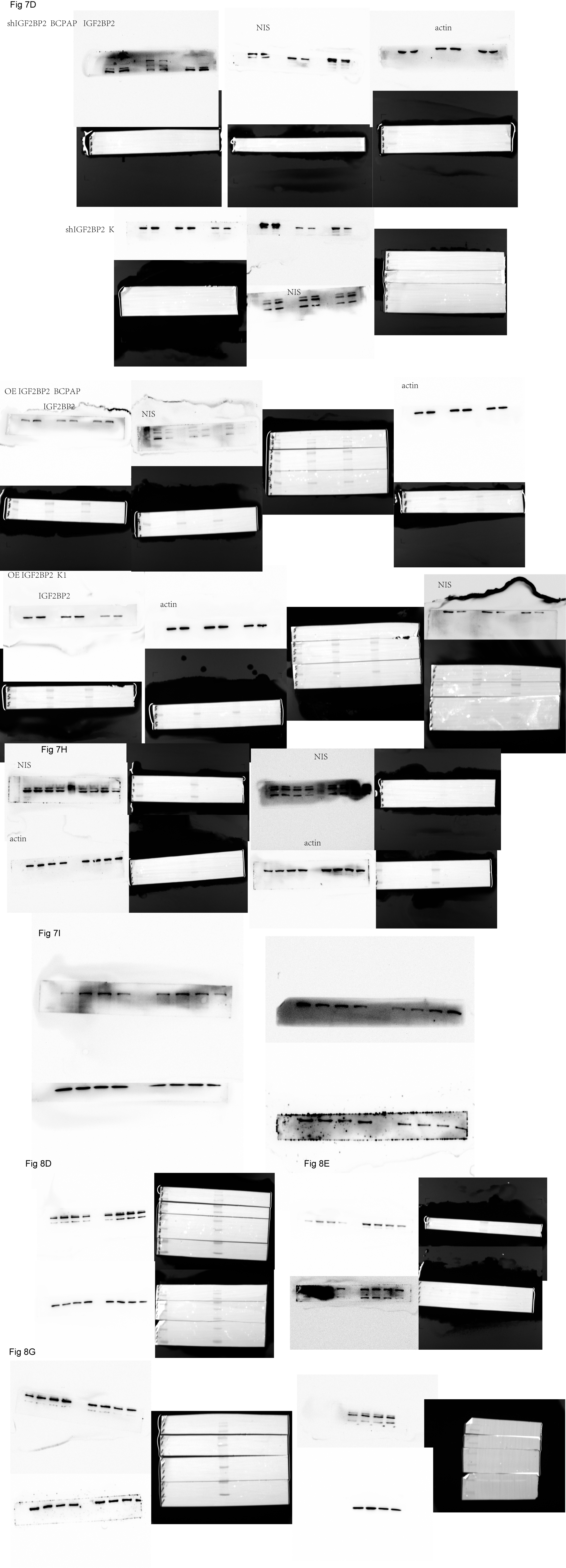

Supplement: Supplementary file 2 [file Image2.TIF]

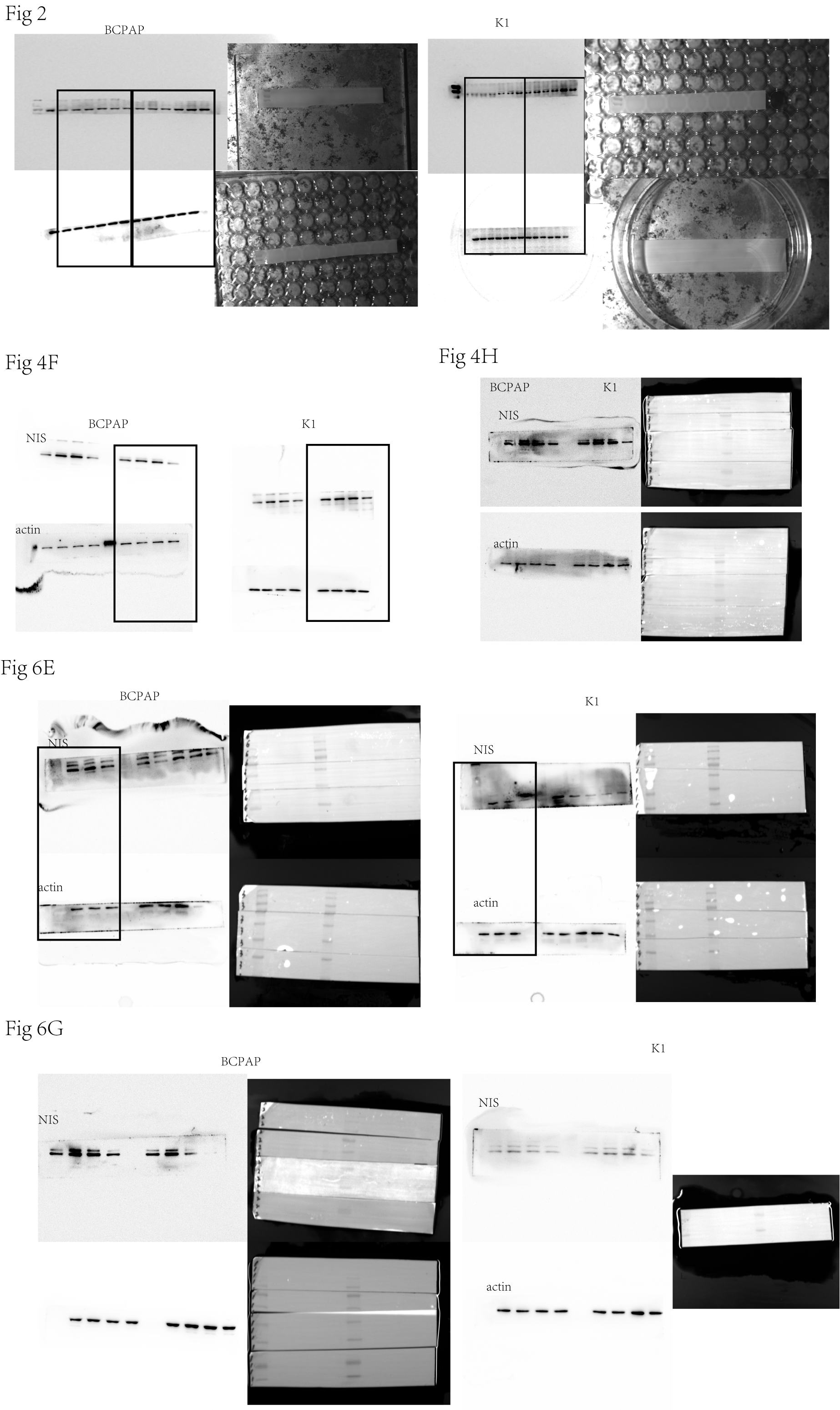

Supplement: Supplementary file 3 [file Image1.TIF]
